# Renal insufficiency caused by *TMEM216* gene mutation: Case Report

**DOI:** 10.3389/fmed.2025.1579732

**Published:** 2025-04-29

**Authors:** Lingjun Sun, Meiqi Xu, Xiaoying Deng, Xiaoyan Liu

**Affiliations:** ^1^Department of Nephrology, The Second Hospital of Dalian Medical University, Dalian, Liaoning, China; ^2^Department of Clinical Laboratory, The Second Hospital of Dalian Medical University, Dalian, Liaoning, China

**Keywords:** chronic kidney disease (CKD), hereditary kidney disease, *TMEM216* gene, ciliopathy, gene mutation, case report

## Abstract

**Introduction:**

Chronic kidney disease (CKD) is a globally prevalent condition characterized by high morbidity and a progressive course that often culminates in end-stage renal disease (ESRD), necessitating dialysis or kidney transplantation. In recent years, genetic factors have received increasing attention in the pathogenesis of CKD, particularly among patients with unexplained renal dysfunction. Genetic screening has emerged as a valuable diagnostic tool. Mutations in the *TMEM216* gene, a pathogenic variant associated with ciliopathy, have been implicated in severe renal impairment. This study presents a case analysis that explores the impact of *TMEM216* mutations on kidney function and their potential clinical significance.

**Case presentation:**

We report a case of a 21-year-old male who developed proteinuria at the age of 15 without an apparent cause. Over the subsequent 6 years, his serum creatinine levels gradually increased, ultimately progressing to ESRD, accompanied by complications such as hypertension and secondary hyperparathyroidism. Imaging studies revealed bilateral renal cysts and a congenital bicuspid aortic valve. Whole-exome sequencing identified compound heterozygous mutations in *TMEM216* [c.253C > T (p.R85*) and c.143 T > C (p.L48P)], consistent with an autosomal recessive inheritance pattern. Family analysis indicated that each parent carried one of the mutations. The combination of clinical and genetic findings suggests that the patient’s renal insufficiency may be attributed to *TMEM216* mutations, highlighting their potential role in the progression of CKD.

**Conclusion:**

This study presents a severe case of renal dysfunction attributed to mutations in the *TMEM216* gene, thereby expanding the clinical phenotypic spectrum associated with this gene. Mutations in ciliopathy-related genes may contribute to proteinuria and renal failure by disrupting the polarization and functionality of renal tubular epithelial cells. For young patients with unexplained CKD, genetic testing can serve as an early diagnostic tool to identify the underlying etiology and facilitate personalized treatment strategies. Future research on *TMEM216*-related nephropathy should aim to further elucidate its pathogenic mechanisms and explore potential therapeutic targets to enhance patient outcomes and advance precision medicine.

## Introduction

Chronic kidney disease (CKD) is a highly prevalent condition worldwide, with a global median prevalence of 9.5% (IQR 5.9–11.7%). According to the Global Burden of Disease (GBD) study, approximately 697.5 million individuals were affected by CKD globally in 2017, and by 2040, CKD is projected to become the fifth leading cause of death worldwide (1, 2). Beyond its high prevalence, CKD imposes a substantial disease burden, as many patients ultimately progress to end-stage renal disease (ESRD), necessitating dialysis or kidney transplantation. The primary etiologies of CKD include diabetes, hypertension, and tubulointerstitial diseases. In addition to these established causes, genetic factors have garnered increasing attention in recent years for their role in the pathogenesis of CKD.

Hereditary kidney diseases represent a heterogeneous group of disorders resulting from gene mutations that impact renal structure and/or function. These conditions typically manifest as progressive kidney dysfunction, which, if left untreated, may lead to CKD or ESRD. Common hereditary kidney disorders include autosomal dominant polycystic kidney disease (ADPKD), Alport syndrome, and various tubulointerstitial nephropathies, each characterized by unique genetic backgrounds and clinical features (3–5).

This case report focuses on a 21-year-old male diagnosed with ESRD. Through genetic testing and an analysis of family history, a diagnosis of hereditary kidney disease due to a mutation in the *TMEM216* gene was established. This case underscores the critical importance of genetic screening in patients with unexplained kidney pathology or a family history of renal disease. Early detection and intervention are vital for slowing disease progression and improving clinical outcomes. The identification of the causal mutation in this patient not only confirmed the hereditary nature of the disease but also offered potential insights into the clinical course and therapeutic targeting of genetically driven kidney disorders. Additional literature regarding *TMEM216* is summarized in Table 1 (6–13).

**Table 1:** Published studies on the role of *TMEM216* in ciliopathies.

Author	Description	Year
Glass et al. (6)	More than 40 genes, including *TMEM216*, have been identified as associated with Joubert syndrome.‌‌	1993
Edvardson et al. (7)	*TMEM216* has been localized to primary cilia and plasma membranes in ciliated cell lines and primary cells, playing a significant role in the apical migration of centrioles and the subsequent formation of primary cilia.	2010
AY Wang (8)	The *TMEM216* gene is crucial for cell polarity, morphogenesis, and ciliogenesis.	2011
Huang et al. (9)	Studies in *Caenorhabditis elegans* revealed that *TMEM216* functions as part of the transition zone (TZ) module, contributing to the spectrum of ciliopathy phenotypes.	2011
Garcia-Gonzalo et al. (11)	Mutations in *TMEM216* and other genes encoding ciliary components cause ciliopathies, indicating that dysfunction in the transition zone is a common pathogenic mechanism underlying these disorders.	2011
Szymanska et al. (10)	*TMEM216* is one of the disease-causing genes for Meckel-Gruber syndrome (MKS).	2012
Gogendeau et al. (12)	Depletion of the *TMEM216* protein in Paramecium can induce persistent ciliary shedding.	2020
Wang et al. (13)	The *TMEM216*-SUFU-GLI2/GLI3 axis plays an essential role in ciliopathies and aberrant Hedgehog (Hh) signaling induced by *TMEM216* deficiency.	2024

## Case report

**Figure 1:**
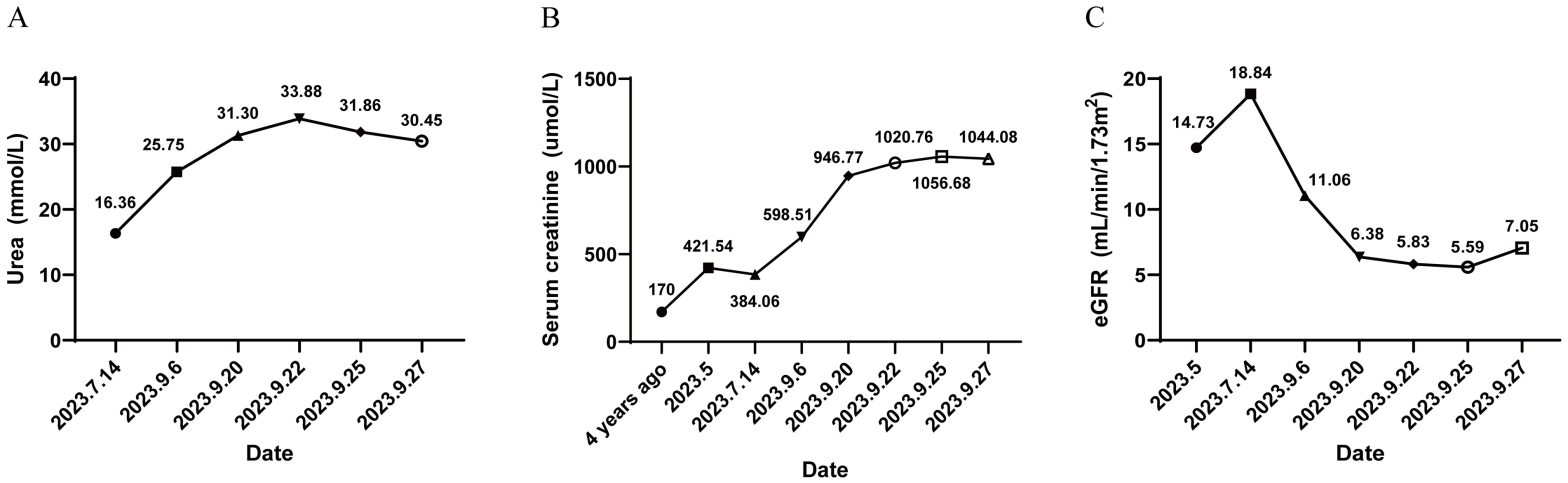
Timeline of renal function-related laboratory tests in the patient, demonstrating their relationship with disease progression and treatment. **(A)** Urea (mmol/L); **(B)** Serum creatinine (μmol/L); **(C)** Estimated glomerular filtration rate (eGFR, mL/min/1.73 m^2^).

**Figure 2:**
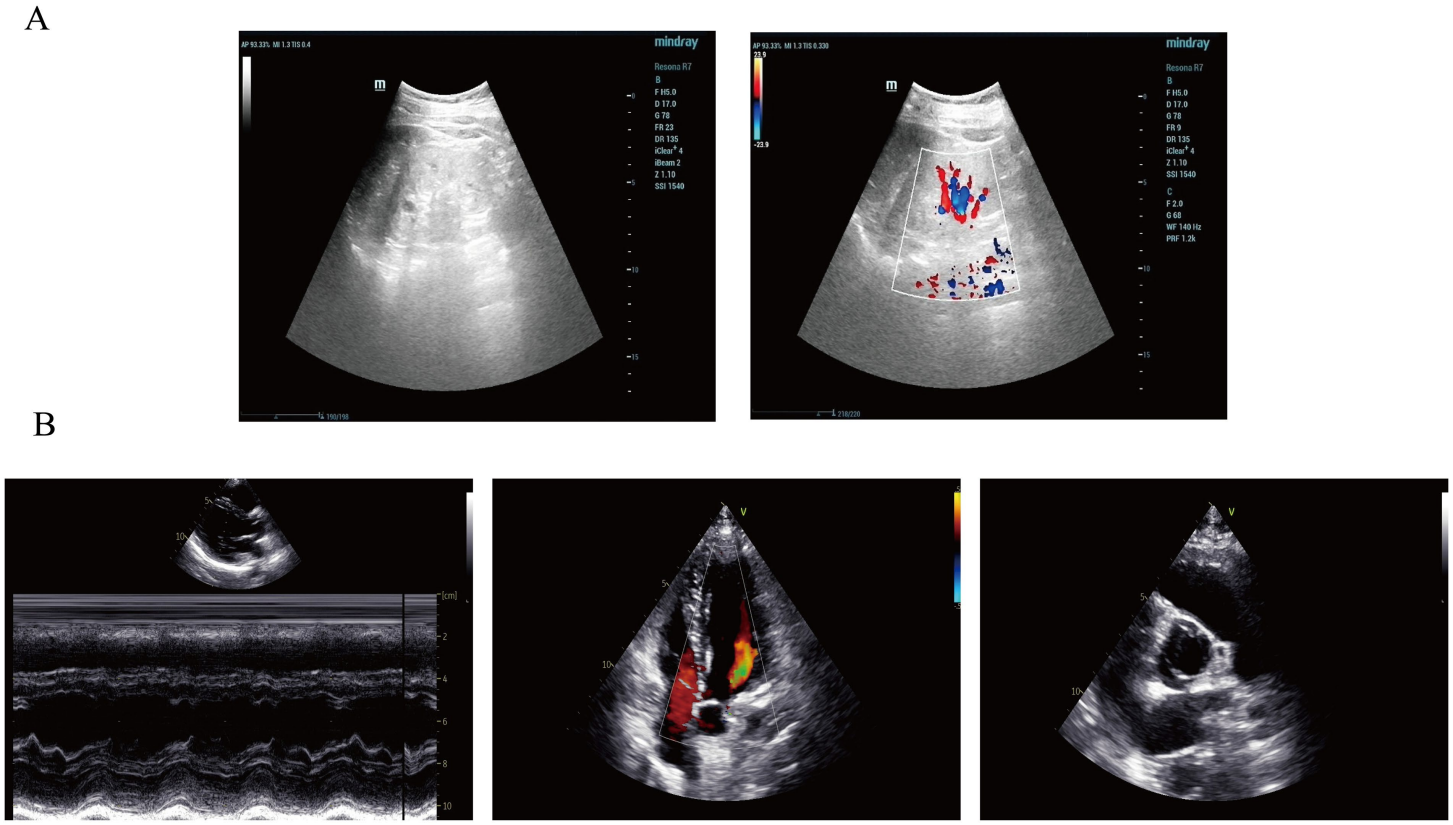
Imaging findings of the patient in this case report. **(A)** Renal ultrasonography revealed multiple anechoic lesions in the renal cortex. In the right kidney, the largest cyst was located at the lower pole, measuring 0.8 × 0.5 cm, with well-defined margins and good internal echogenicity. In the left kidney, multiple cortical cysts were observed, with the largest measuring 1.0 × 0.8 cm in the mid-region, showing clear margins and good posterior acoustic enhancement. These findings are consistent with bilateral renal cysts. **(B)** Color Doppler echocardiography (CDE) demonstrated normal sizes of all cardiac chambers, with no thickening of the interventricular septum or left ventricular wall, and preserved wall motion. The aortic valve showed fusion of the left and right coronary cusps, arranged in a right–left orientation with a commissural angle of approximately 160°. A fusion ridge was visible between the fused cusps, with good valve opening and a commissural slit, indicating a congenital bicuspid aortic valve (right–left, Type I). The morphology and structure of the remaining valves appeared normal.

A 21-year-old male presented with proteinuria that first manifested 6 years ago without an identifiable cause. At that time, no further medical assessment or treatment was pursued. Four years ago, an evaluation of renal function at an external hospital revealed an elevated serum creatinine level of 170.00 μmol/L. Subsequent follow-up indicated a gradual increase in serum creatinine levels. He intermittently took Haikun Shenxi Capsules (two capsules, three times daily), a traditional Chinese medicine preparation primarily composed of fucoidan sulfate polysaccharide, which is clinically utilized for the treatment of chronic renal failure. Its reported therapeutic effects include improvement of renal blood flow, attenuation of glomerulosclerosis, promotion of renal function recovery, scavenging of reactive oxygen species, and inhibition of lipid peroxidation (14). Four months prior to admission, the patient’s serum creatinine level rose to 421.54 μmol/L, and his hemoglobin level was recorded at 113.00 g/L. Roxadustat was subsequently introduced to address renal anemia. Two weeks before admission, the patient experienced bone pain accompanied by pruritus. Laboratory tests revealed a parathyroid hormone (PTH) level of 627.50 pg./mL, urea 25.75 mmol/L, creatinine 598.51 μmol/L, and estimated glomerular filtration rate (eGFRcr) of 11.06 mL/min/1.73 m^2^. Serum inorganic phosphorus was 1.55 mmol/L. He was treated with sevelamer and calcitriol, which led to partial relief of pruritus and bone pain. However, renal function deteriorated significantly. Ten days prior to the latest evaluation, serum urea increased to 31.30 mmol/L and creatinine to 946.77 μmol/L, with an eGFRcr of 6.38 mL/min/1.73 m^2^. Urinalysis revealed “++” proteinuria, with a 24-h urine protein excretion of 1220.67 mg/24 h. PTH level was 253.10 pg./mL (Figure 1). B-mode ultrasonography of the urinary system revealed multiple anechoic lesions in the bilateral renal cortices, consistent with bilateral renal cysts (Figure 2A). The patient had a history of hypertension lasting over 4 years, with maximum readings of 140–150/100–90 mmHg. Initially, blood pressure was managed with valsartan capsules; however, due to suboptimal control, the treatment was switched to benidipine, resulting in improved blood pressure stability. Additionally, the patient had a previous diagnosis of hypothyroidism and was regularly taking levothyroxine at a dosage of half to three-quarters of a tablet daily. Transthoracic color Doppler echocardiography (CDE) revealed a bicuspid aortic valve (type I, R/L), characterized by the fusion of the right and left coronary cusps arranged in a left–right orientation, with a commissural angle of approximately 160°. A fused ridge was noted between the fused leaflets, which opened well but exhibited a visible raphé (Figure 2B). Chest and upper abdominal CT scans no significant abnormalities. During the evaluation, the patient exhibited mildly delayed cognitive development compared to peers. However, no overt dysmorphic features or neurological signs were observed.

**Figure 3:**
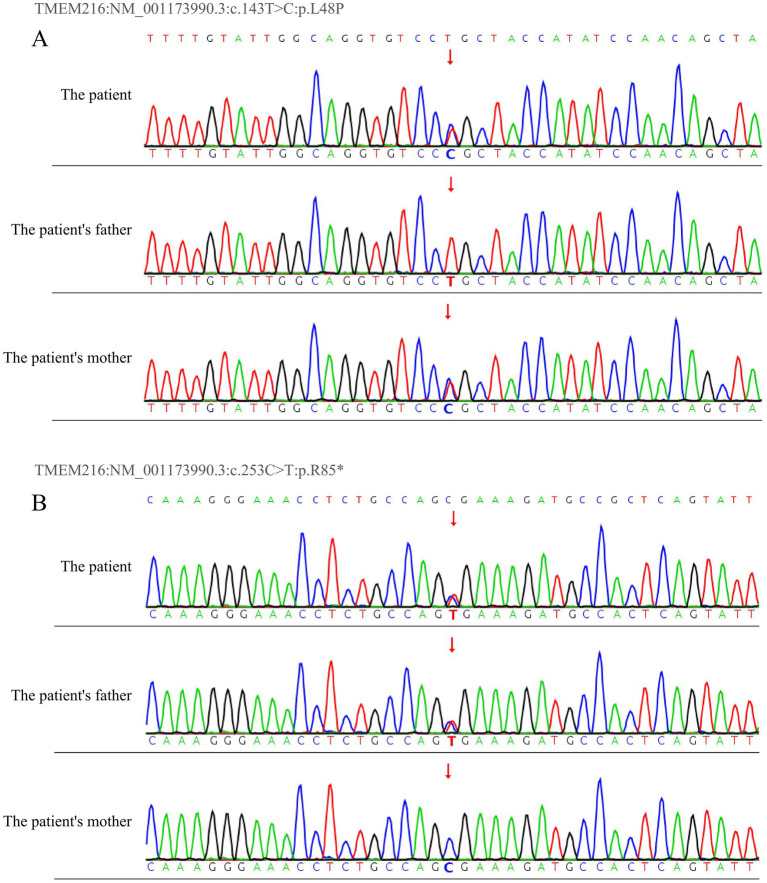
DNA sequencing results of *TMEM216* variants in the patient’s family. Sanger sequencing of the *TMEM216* gene revealed **(A)** the patient and his mother both carry the heterozygous variant c.143 T > C (p.L48P), and **(B)** the patient and his father both carry the heterozygous variant c.253C > T (p.R85*).

**Figure 4:**
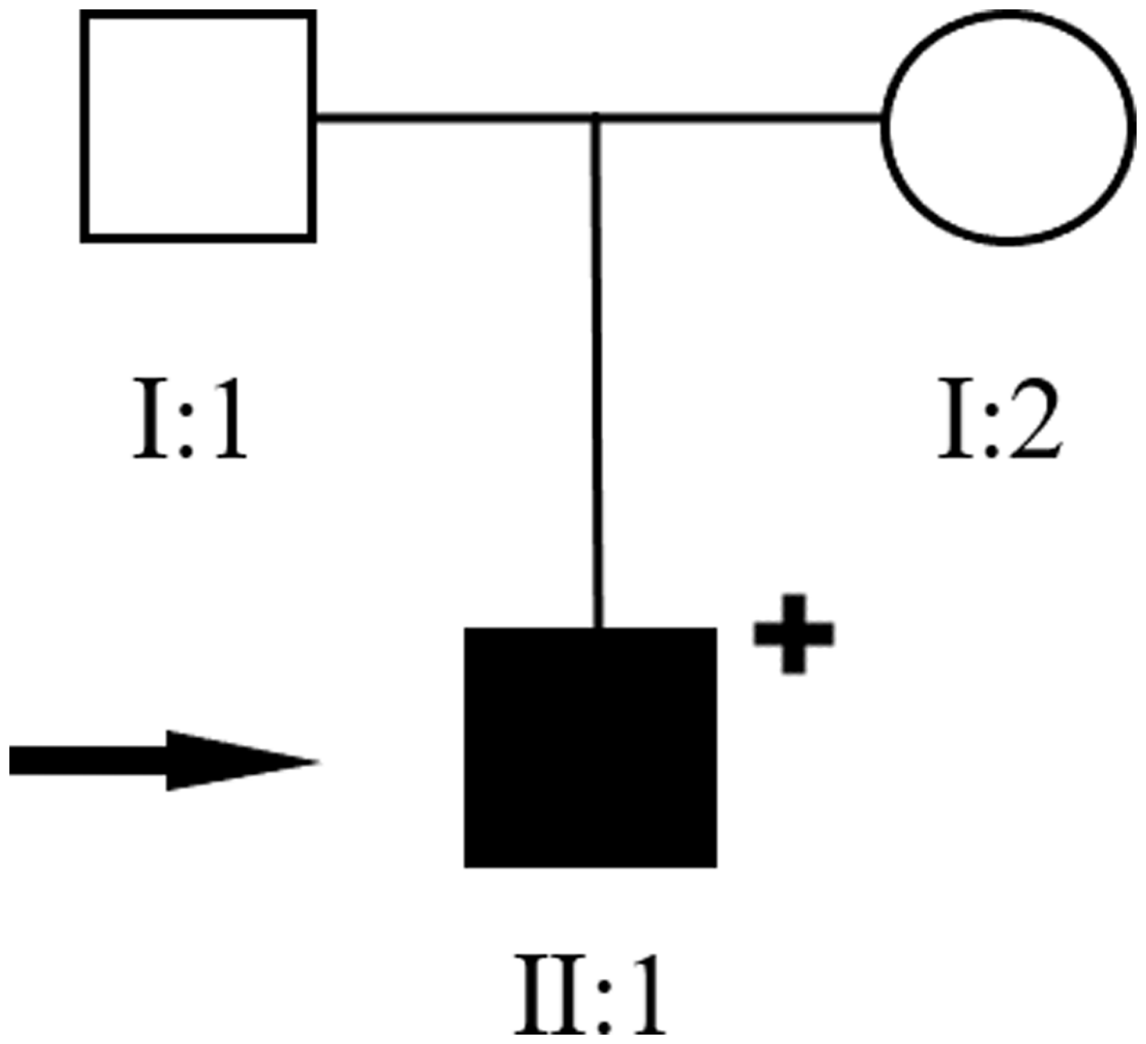
Pedigree of the reported family. Filled black symbols indicate individuals affected by hereditary kidney disease. The arrow denotes the proband carrying compound heterozygous mutations in exons 3 and 4 of the *TMEM216* gene (clinical details provided in the main text).

The patient is a young male presenting with mild intellectual developmental difficulties. CDE indicated a congenital bicuspid aortic valve malformation, and a hereditary condition could not be excluded. To investigate the potential genetic etiology underlying the onset and progression of the disease, whole-exome sequencing (WES) was performed on the patient and his parents. Sequencing was conducted on the Illumina NovaSeq 6,000 platform, and raw reads were aligned to the human reference genome (hg38/GRCh38) using Burrows-Wheeler Aligner (BWA). Variant calling was subsequently carried out using the Verita Trekker® variant detection system and GATK, followed by variant annotation and interpretation using ANNOVAR and the Enliven® annotation system (15). According to the guidelines and recommendations from the American College of Medical Genetics and Genomics (ACMG), and after filtering based on inheritance pattern, age of onset, population frequency, and pathogenicity predictions, compound heterozygous variants were identified in the *TMEM216* gene of the patient: c.253C > T (p.R85*) and c.143 T > C (p.L48P). The inheritance of these variants was validated by Sanger sequencing. The results showed that both the patient and his father carried the heterozygous c.253C > T (p.R85*) variant, which results in a cytosine (C) to thymine (T) substitution at nucleotide position 253 of the cDNA, creating a premature stop codon at amino acid position 85 (p.R85*). Meanwhile, the patient and his mother both carried the heterozygous c.143 T > C (p.L48P) variant, which leads to a thymine (T) to cytosine (C) substitution at nucleotide position 143, resulting in a leucine (Leu) to proline (Pro) substitution at position 48 of the protein (p.L48P). The c.253C > T (p.R85*) variant has been previously reported as pathogenic in studies related to Meckel syndrome and other ciliopathies, leading to premature stop codon formation. This variant showed co-segregation with the disease phenotype in two affected family members (10). Fulfilling the ACMG criteria of PVS1 + PM3 + PP1 + PM2_Supporting. It has been classified as pathogenic (P) in the ClinVar database and as a disease-causing mutation (DM) in the HGMD database (16, 17). This pathogenic variant is known to cause loss of protein function and is associated with severe ciliopathy phenotypes. In contrast, the c.143 T > C (p.L48P) variant has not been previously reported in the literature, and no functional studies or familial segregation data are available to support its pathogenicity. Based on the PM3 + PM2_Supporting criteria, it was classified as a variant of uncertain significance (VUS) (Figure 3). Interestingly, although the patient’s father also carries the pathogenic c.253C > T (p.R85*) variant in *TMEM216*, he does not exhibit any clinical renal phenotype, suggesting that the additional c.143 T > C (p.L48P) variant may play a contributory role in the manifestation of renal disease in the patient. The inheritance pattern observed in this family is consistent with autosomal recessive inheritance (Figure 4).

## Discussion

Hereditary kidney diseases are prevalent across various populations, with multiple common genetic variants have been confirmed to be closely associated with the onset of IgA nephropathy (IgAN), membranous nephropathy, and nephrotic syndrome (18–21). Consequently, the pathogenesis of most kidney diseases is no longer regarded as driven by a single factor but rather results from the combined influence of various genetic and environmental contributors that collectively promote disease initiation and pathological progression. In this report, we present a male patient with hereditary renal insufficiency caused by mutations in the *TMEM216* gene, which ultimately led to ESRD. The WES of the patient and his parents revealed a heterozygous nonsense mutation in exon 4 of *TMEM216* inherited from the father, and a heterozygous missense mutation in exon 3 inherited from the mother. Both mutations were identified in the patient.

*TMEM216* encodes a tetraspan transmembrane protein located in the ciliary transition zone. By interacting with other cilia-associated proteins, *TMEM216* plays a role in regulating the formation and localization of primary cilia (22). Primary cilia are critical for kidney organogenesis, particularly in maintaining the differentiation and proliferation of epithelial cells (23). Previous studies have shown that deletion of the ciliary regulatory gene *TMEM16A* in murine nephrons leads to a reduced number of primary cilia in proximal tubular cells, accompanied by increased proteinuria. This suggests that a reduction in cilia number can disrupt the polarity and function of tubular epithelial cells, thereby impairing the reabsorptive capacity of renal tubules—especially the reabsorption of low-molecular-weight proteins (24, 25).

Studies have demonstrated that knockdown of *TMEM216* impairs ciliogenesis in polarized cells and disrupts proper docking of the centrosome at the apical cell surface (26). Mutations in *TMEM216* are closely associated with disorders such as Joubert syndrome and Meckel syndrome, which are characterized by multisystem abnormalities including neurodevelopmental defects, renal anomalies, and retinal degeneration (Table 2) (6, 27–31). Among these manifestations, renal abnormalities typically include renal cysts, renal insufficiency, and cystic renal dysplasia. Clinically, patients with Joubert or Meckel syndrome may present with progressive decline in renal function, suggesting that *TMEM216* mutations may contribute to renal pathologies in these syndromes by impairing ciliogenesis (32).

**Table 2:** Clinical features of Joubert syndrome and Meckel syndrome.

System organ classification (SOC)	Joubert syndrome	Meckel syndrome
Nervous system	Brainstem hypoplasia, vermian hypoplasia of the cerebellum, ataxia, hypotonia, developmental delay, epilepsy, oculomotor disturbances, and hydrocephalus.	Occipital encephalocele, craniorachischisis, partial corpus callosum agenesis, dandy-walker malformation, anencephaly.
Ocular	Nystagmus, ocular motility disturbances, retinal dystrophy, optic nerve atrophy, visual impairment, hypertelorism, choroidal retinal defects, optic disc anomalies.	Retinal coloboma, congenital microphthalmia
Facial and craniofacial features	Mild craniofacial anomalies	Cleft lip, cleft palate
Respiratory system	Neonatal respiratory abnormalities (alternating periods of rapid and slow breathing), apnea.	Pulmonary hypoplasia
Renal system	Renal structural abnormalities (such as renal hypoplasia), tubulointerstitial disease, and Renal cysts.	Bilateral cystic dysplasia of the kidneys, kidney enlargement, and corticomedullary differentiation.
Hepatobiliary system	Liver fibrosis, cholestasis, and liver dysfunction.	Bile duct proliferation and dilation, hepatic fibrosis, hepatic cysts
Skeletal system	Polydactyly and spinal deformities	Polydactyly, bowed and shortened long bones
Reproductive system	Undescended testes and external genitalia anomalies	Hypoplasia of male genitalia
Psychiatric and behavioral disorders	Autistic-like features, attention deficit, cognitive impairments, inattention, hyperactivity, stereotypies, emotional instability, anxiety, self-harm, aggression, and autism	Intellectual disability, developmental delay
General symptoms/systemic	Developmental delay, growth retardation	Growth restriction
Other manifestations	Hearing loss (sensorineural), feeding difficulties, gastroesophageal reflux	Cystic hygroma

In the present case report, the patient developed proteinuria at the age of 15 without any obvious cause, followed by a gradual increase in serum creatinine over 6 years, ultimately progressing to ESRD accompanied by secondary hyperparathyroidism and other related complications. Ultrasonographic imaging revealed bilateral renal cysts and a congenital bicuspid aortic valve malformation. Whole-exome sequencing identified compound heterozygous mutations in the *TMEM216* gene (c.253C > T (p.R85*) and c.143 T > C (p.L48P)). From a clinical perspective, the pathogenesis of renal insufficiency in this patient can be interpreted within the framework of ciliopathy-related mechanisms. Defects in primary cilia impair the ability of renal tubular epithelial cells to sense urinary flow, resulting in dysregulation of electrolyte metabolism, particularly calcium, which contributes to the development of proteinuria and progressive renal dysfunction. Genetically, both the patient and his parents (with no known consanguinity) were found to carry *TMEM216* mutations, with the patient exhibiting compound heterozygosity. This finding supports an autosomal recessive inheritance pattern. The *TMEM216* mutations likely impair ciliogenesis and ciliary function, leading to secondary injury of the renal tubules and contributing to the onset and progression of CKD.

The current therapeutic principles for *TMEM216* mutation–associated renal insufficiency primarily include delaying disease progression, renal replacement therapy (RRT), and reducing the risk of complications. In early-stage patients, cAMP modulators or mTOR inhibitors may be considered to slow the progression of cystic lesions (33), while closely monitoring renal function deterioration. For patients who have progressed to ESRD, peritoneal dialysis (PD) is often the preferred modality, especially in younger individuals or those with significant neurological involvement; hemodialysis serves as an alternative when PD fails or is not tolerated. Kidney transplantation remains the optimal option for improving long-term outcomes, but comprehensive evaluation of hepatic and neurological comorbidities is essential to ensure the feasibility and safety of transplantation (34). In addition, gene therapy approaches such as antisense oligonucleotide (ASO) technology have shown promising results in reducing cyst formation in CEP290-related renal disease models (35) and may represent a potential precision treatment strategy for *TMEM216*-related kidney disorders in the future. *TMEM216* mutations are typically associated with severe renal phenotypes, often accompanied by central nervous system abnormalities or syndromic manifestations. However, most previously reported *TMEM216* mutations have rarely presented with isolated renal involvement (7, 26, 36). In our reported case, the patient exhibited primarily renal phenotypes—renal cysts and renal failure—alongside intellectual developmental difficulties, highlighting the possibility that *TMEM216* mutations can manifest predominantly as renal disease. For young patients with unexplained CKD, it is necessary to perform exome sequencing or targeted gene testing to identify potential hereditary causes early. Moreover, individuals carrying pathogenic variants should receive genetic counseling and undergo longitudinal follow-up based on their renal function status to guide long-term management. Early diagnosis and personalized interventions can effectively slow disease progression, reduce complications, and improve overall prognosis.

Meanwhile, further investigations into the role of *TMEM216* in kidney diseases, particularly in tubular injury, interstitial fibrosis, and other ciliopathies—may provide critical insights into the molecular mechanisms underlying renal pathophysiology and reveal novel therapeutic targets.

## Data Availability

The original contributions presented in the study are included in the article/supplementary material, further inquiries can be directed to the corresponding author.
